# Complement-Activating IgM Enhances the Humoral but Not the T Cell Immune Response in Mice

**DOI:** 10.1371/journal.pone.0081299

**Published:** 2013-11-08

**Authors:** Zhoujie Ding, Anna Bergman, Christian Rutemark, Rika Ouchida, Hiroshi Ohno, Ji-Yang Wang, Birgitta Heyman

**Affiliations:** 1 Department of Medical Biochemistry and Microbiology, Uppsala University, Uppsala, Sweden; 2 Laboratory for Immune Diversity, RIKEN Research Center for Allergy and Immunology, Tsurumi, Yokohama, Japan; 3 Laboratory for Intestinal Ecosystem, RIKEN Center for Integrative Medical Sciences, Tsurumi, Yokohama, Japan; 4 Department of Immunology, Medical Research Institute, Tokyo Medical and Dental University, Tokyo, Japan; 5 Department of Immunology, Shanghai Medical College, Fudan University and Biotherapy Research Center of Fudan University, Shanghai, China; Johannes Gutenberg University of Mainz, Germany

## Abstract

IgM antibodies specific for a certain antigen can enhance antibody responses when administered together with this antigen, a process believed to require complement activation by IgM. However, recent data show that a knock-in mouse strain, Cμ13, which only produces IgM unable to activate complement, has normal antibody responses. Moreover, the recently discovered murine IgM Fc receptor (FcµR or TOSO/FAIM3) was shown to affect antibody responses. This prompted the re-investigation of whether complement activation by specific IgM is indeed required for enhancement of antibody responses and whether the mutation in Cµ13 IgM also caused impaired binding to FcµR. The results show that IgM from Cµ13 and wildtype mice bound equally well to the murine FcµR. In spite of this, specific Cμ13 IgM administered together with sheep red blood cells or keyhole limpet hemocyanine was a very poor enhancer of the antibody and germinal center responses as compared with wildtype IgM. Within seconds after immunization, wildtype IgM induced deposition of C3 on sheep red blood cells in the blood. IgM which efficiently enhanced the T-dependent humoral immune response had no effect on activation of specific CD4^+^ T cells as measured by cell numbers, cell division, blast transformation, or expression of the activation markers LFA-1 and CD44 in vivo. These observations confirm the importance of complement for the ability of specific IgM to enhance antibody responses and suggest that there is a divergence between the regulation of T- and B-cell responses by IgM.

## Introduction

Antibodies, passively administered together with antigen, can dramatically alter the immune response to the antigen via antibody feedback regulation. The effects are antigen specific and can lead to more than 99% suppression or to several hundred-fold enhancement depending on the type of antigen and antibody isotype (reviewed in 1). IgG is able to suppress responses to large antigens such as erythrocytes, and this has been used successfully in the clinic since the 1960's to prevent hemolytic disease of the newborn [2,3]. Rhesus negative mothers carrying Rhesus positive babies can become immunized after transplacental hemorrage and produce IgG anti-RhD which will damage fetal erythrocytes. This immunization can be prevented by administration of preformed IgG anti-RhD to the mothers. In contrast, administration of IgM anti-RhD together with Rhesus positive erythrocytes leads to (unwanted) higher antibody responses, illustrating that IgM is able to feedback enhance the immune response to erythrocytes [2]. 

Most studies of the mechanism behind IgM-mediated enhancement have been done in mouse models using sheep red blood cells (SRBC) [4-8] or the large protein keyhole limpet hemocyanine (KLH) [9,10] as model antigens. IgM rarely enhances responses to smaller proteins and can only enhance responses to suboptimal antigen doses [4]. The enhancement is antigen- but not epitope-specific, i e IgM specific for one determinant on SRBC enhances responses also to other determinants even if they are not recognized by the passively administered IgM [6,9,11]. IgM cannot enhance antibody responses in T cell deficient nude mice, and thus does not substitute for T cell help [12]. IgM-mediated enhancement is thought to depend on the ability of IgM to activate complement. This conclusion is based on two sets of experiments. First, mutant monoclonal IgM which, owing to a point mutation in the Cμ heavy chain, had lost the ability to bind C1q, also lost the ability to enhance antibody responses [11]. Second, monomeric IgM, which does not activate complement, failed to enhance antibody responses [10]. 

A connection between complement and antibody responses was first made in the classical experiments where depletion of C3 by cobra venom factor led to impaired antibody responses [13]. Subsequently, it was found that only classical pathway components are required, since C1q knock-out mice [14,15], but not mice lacking alternative or lectin pathway factors [16,17], had impaired antibody responses. This suggested that antibodies, being the most efficient classical pathway activators, were involved at the onset of antibody responses by forming immune complexes and binding C1q. The requirement for C1q not only for secondary, but also for primary antibody responses [14,15] (reviewed in 18), seemed like a paradox since very little specific antibodies are present in a naive animal. A possible solution presented itself when natural IgM was shown to play a role for the generation of primary antibody responses [19,20]. This suggested that natural IgM would bind antigen, activate complement and trigger an early response resulting in production of specific IgM which would further enhance the antibody response via the feedback pathways described above. This idea was recently tested in knock-in mice (Cμ13), constructed to have the same point mutation in their Cμ heavy chain as did the mAb used to show that loss of C1q binding also led to loss of ability to enhance antibody responses [11]. Surprisingly, antibody responses to SRBC and KLH were normal in Cμ13 mice, although all their IgM molecules were unable to activate complement [15]. In addition, recent studies revealed that the IgM Fc receptor (FcμR) is required for efficient humoral immune responses, especially when the amount of antigen is limited [21,22]. These observations prompted us to re-investigate whether the immunoenhancing effect of specific IgM is indeed complement dependent. As mentioned, this conclusion was based on the lack of enhancement by mutant IgM as well as by monomeric IgM, both unable to activate complement [10,11]. However, these changes in the IgM molecule may also have led to impaired binding to FcμR. Moreover, the failure of the mutant IgM to enhance was demonstrated with one single TNP (trinitrophenyl)-specific mAb with a weak enhancing capacity, possibly owing to low affinity [11]. The Cμ13 strain made it possible to obtain mutant IgM with high titers against any desired antigen and to study its ability to enhance antibody responses. Cloning of the FcμR enabled transfection of cell lines and analysis of the binding by Cμ13 and WT (wildtype) IgM. Our results show that Cμ13 and WT IgM bound equally well to FcμR, but that only WT IgM was an efficient enhancer of humoral responses.

## Materials and Methods

### Ethics statement

Animal experiments were approved by Uppsala Animal Research Ethics Committee (Permit numbers: C146/10 and C25/13). The mice were bred and maintained in the animal facilities at the National Veterinary Institute (Uppsala, Sweden). Skilled personnel under the supervision of the veterinarian in charge routinely observed the health status of the mice.

### Mice

BALB/c mice (referred to as WT) were from Bommice (Ry, Denmark). Cμ13 mice, backcrossed to BALB/c for 12 generations, produce IgM with a point mutation in position 436 in the μ heavy chain making it unable to activate complement [15]. DO11.10 mice on a BALB/c background, carrying a transgenic T cell receptor (TCR) recognizing peptide 323-329 of ovalbumin (OVA) together with MHC class II I-A^d^ [23], were obtained from Dr Lisa Westerberg (Karolinska Institute, Stockholm). Mice were matched for age and sex within each experiment.

### Antigens

KLH, OVA, and picrylsulfonic acid/hydrate (as a source of TNP) were purchased from Sigma Aldrich (St Louis, MO, USA). SRBC were obtained from the National Veterinary Institute (Håtunaholm, Sweden) and stored sterile at 4°C in Alsever’s solution. SRBC were washed three times in PBS before use. OVA was conjugated to SRBC, and the conjugation was confirmed by flow cytometry [24]. OVA-TNP conjugation was performed as described [25] and the number of TNP residues/OVA determined [25]. A batch with 2.4 TNP residues/OVA was used.

### Isolation of antigen-specific IgM, IgG, and IgE

BALB/c and Cμ13 mice were immunized i.v. with 0.2 ml of a 10% SRBC suspension or 100 μg KLH in PBS and bled five days later. IgM anti-KLH and IgM anti-SRBC were obtained by size fractionation of sera over a Sepharose-CL 6B column (GE-Healthcare, Uppsala, Sweden), dialyzed, concentrated, steril filtered and stored at 4°C [8]. IgM anti-SRBC was ultracentrifuged at 150,000 g for 150 min to remove aggregates in experiments where antibody responses were measured. Rabbit IgG anti-SRBC was purified from hyperimmune sera with a protein A Sepharose column (GE Healthcare) as described earlier [26]. Monoclonal IgE anti-TNP antibodies were derived from IGELb4 hybridomas [27], purified and stored as described [28].

### Hemagglutination and hemolysis assays

Hemagglutination and hemolysis assays were performed as described [6,8]. The hemagglutination titer (HA) of I gM anti-SRBC was defined as the highest dilution in PBS able to agglutinate 0.25% SRBC at 37 °C after 1 h incubation. Hemolysis assays were performed in Hank's balanced salt solution (BSS) with guinea pig serum (diluted 1:256) as a source of complement. The hemolytic titer of IgM anti-SRBC was defined as the highest dilution completely lysing 0.25% SRBC at 37 °C after 1 h incubation.

### Immunofluorescence staining in flow cytometry and confocal microscopy

For flow cytometry: rat IgG2bκ anti-CD16/CD32 (clone 2.4G2; BD Biosciences, San José, GA, USA) was used as Fc-block. Rabbit polyclonal IgG anti-SRBC (purified as above), Alexa Fluor 647-labeled goat anti-rabbit IgG (Life technologies, Grand Island, NY, USA) and fluorescein isothiocyanate (FITC)-labeled rat IgG2a anti-C3 (CL7503F; Cedarlane, Birlington, Ontario, Canada) were used to detect C3 deposition on SRBC. Alexa Fluor 647-labeled rat IgMκ anti T- and B-cell activation antigen (clone GL7, BD Biosciences), pacific blue-labeled rat IgG2aκ anti-CD45R (B220) (clone RA3-6B2; eBioscience, San Diego, CA, USA) and biotinylated peanut agglutinin (PNA; Vector Laboratories, Burlingame, CA, USA) followed by FITC-labeled streptavidin (BD Pharmingen, San Diego, CA, USA) were used to identify germinal center B cells. FITC-labeled mouse IgG2a anti-DO11.10 TCR (clone KJ1-26; Life technologies) or biotinylated mouse IgG2a anti-DO11.10 TCR (clone KJ1-26; eBioscience) followed by allophycocyanin (APC)-conjugated streptavidin (eBioscience) and phycoerythrin (PE)-labeled rat IgG2bκ anti-CD4 (GK1.5; BD Biosciences) were used for detecting OVA-specific CD4^+^ cells. FITC-labeled rat IgG2aκ anti-CD11a/LFA-1 (clone I21/7; Life technologies) and FITC-labeled rat IgG2b anti-CD44 (IM7.8.1; Life technologies) were used for detecting T cell activation. To confirm FcμR expression on transfected cells, rat IgG2a anti-FcμR (clone 4B5) [21] or an isotype control antibody (clone eBR2a; eBioscience), followed by PE-labeled anti-rat IgG2a (clone RG7/1.30; BD Biosciences) were used. The binding of WT and Cμ13 IgM to the mouse FcμR was confirmed with biotinylated anti-IgM antibody (clone eB121-15F9; eBioscience), followed by streptavidin-conjugated PE (SA-PE; eBioscience). For confocal microscopy: FITC-labeled rat IgG2aκ anti CD169 (clone MOMA-1; AbD Serotec, Oxford, UK), pacific blue-labeled rat IgG2aκ anti-CD45R (clone RA3-6B2; eBioscience) and biotinylated PNA (Vector Laboratories) together with SA-PE (eBioscience) were used to detect germinal centers.

### Immunizations and blood sampling

Mice were immunized intravenously (i.v.) with indicated antibody and antigen doses diluted in 0.2 ml PBS. IgM was given 30 min prior to the antigen. IgE and antigen were pre-mixed before immunization. For investigating C3-deposition on SRBC, mice were bled from the tail artery in tubes containing 1μl lepirudin (50 mg/ml, Refludan, Celgene AB, Summit, NJ, USA) 1 min after immunization, and kept on ice [29]. Lepirudin is an anticoagulant which does not activate complement [30]. For measuring antibody responses, mice were bled from the tail artery at indicated time points and sera were stored at -20 °C before analysis.

### Enzyme-linked immunosorbent assay (ELISA)

The IgG anti-KLH, anti-SRBC and anti-OVA ELISAs were performed as described previously using alkaline phosphatase conjugated sheep anti-mouse IgG (Jackson ImmunoResearch Laboratories, West Grove, PA) [15,24]. The absorbance at 405 nm was read after 30 min incubation with substrate and data were analyzed with SOFTmax software (Molecular Devices, Sunnyvale, CA, USA). Results are given as ng/ml or μg/ml after calculations based on a standard curve using hyperimmune mouse polyclonal IgG anti-SRBC or anti-OVA. In the anti-KLH ELISA, sera were diluted 1:125 and data were shown as absorbance.

### Flow cytometry

Blood samples were diluted 1:50 in FACS buffer (PBS with 2% fetal bovine serum, Sigma-Aldrich). Single cell suspensions from spleens were treated with hypotonic buffer (0.15 M NH_4_Cl, 10mM KHCO_3_, 1 mM EDTA, pH 7.3) for 3 min on ice to lyse erythrocytes, washed once in PBS and resuspended in 3 ml of FACS buffer. Fifty microliter of diluted blood sample or 10^6^ splenocytes were first incubated with Fc block (BD Biosciences) in 100 μl FACS buffer for 5 min at 4 °C and then stained with predetermined optimal amounts of antibodies for 30 min at 4 °C. For each sample, 4-8×10^5^ events were acquired on a FACScan cytometer (BD Biosciences) or a LSR II cytometer (BD Biosciences) at the BioVis platform, SciLifeLab, Uppsala. Red blood cell and lymphocyte populations were gated according to forward- and side-scatter properties. To analyze binding of WT and Cμ13 IgM to the mouse FcμR, BW5147 cells expressing mFcμR-IRES-GFP or GFP alone, as well as virus nontransduced BW5147 cells, were first incubated with SRBC-specific WT IgM, or Cμ13 IgM, or with buffer alone as a control (non-staining). After washing, the cells were incubated with anti-IgM antibody followed by SA-PE (eBioscience). All flow cytometry data were analyzed with FlowJo software (Tree Star Inc., Ashland, OR, USA).

### Confocal Microscopy

Spleens were removed and flash-frozen in optimal cutting temperature (OCT) embedding media (Sakura Finetek, Alphen aan den Rijn, The Netherlands). Eight-micrometer sections were cut with a cryostat, thaw-mounted on frost plus glass slides (Menzel-Gläser, Braunschweig, Germany), air-dried and stored in -80 °C until use. Slides were fixed in 4% paraformaldehyde (Merck, Darmstadt, Germany) in PBS (pH 7.8) for 15 min and then blocked with 5% horse sera (Sigma-Aldrich) for 30 min. Slides were stained with primary antibodies for 1h. After washing twice in PBS, SA-PE was added for 1h. Slides were then washed twice in PBS before mounting in Fluoromount G (Southern Biotech, Birminghan, AL, USA) and analyzed with an LSM 700 confocal microscope (Carl Zeiss, Thornwood, NY, USA). Tile-scan images of each whole section were taken by Zen 2009 software (Carl Zeiss) and processed with ImageJ software (NIH, Bethesda, MD, USA). Percentages of PNA^+^ follicles were quantified from 2-3 non-consecutive sections for each sample.

### Establishment of the mouse FcμR stable transductants

A 1.3-kb mouse *FcμR* cDNA (mFcμR) fragment was cloned into pMX-IRES-GFP retroviral vector [31] to generate mFcμR-IRES-GFP. This retroviral construct and the control pMX-IRES-GFP (GFP) vector were each transfected into the PHOENIX packaging cell line, and the virus supernatant was prepared as described previously [31]. The mouse T cell leukemia cell line BW5147 was transduced with retrovirus expressing mFcμR-IRES-GFP or GFP alone as described [32] and the GFP^+^ cells were sorted 2 days after virus transduction. The sorted cells were expanded and analyzed for FcμR expression by flow cytometry analysis as described [32]. Briefly, the cells were first incubated with Fc block and then stained with either anti-FcμR or an isotype control antibody followed by phycoerythrin (PE)-conjugated anti-rat IgG2a (clone RG7/1.30; BD Biosciences). The BW5147 cells not transduced with retrovirus were included as a control.

### Analysis of WT and Cμ13 IgM binding to the mouse FcμR

10^6^ BW5147 cells expressing mFcμR-IRES-GFP or GFP alone as well as virus nontransduced BW5147 cells were first incubated with 50 μl (final concentration 25 μg/ml) of WT or Cμ13 IgM anti-SRBC antibodies, or with buffer alone as a control (non-staining) on ice for 30 min. After washing with FACS buffer three times, the cells were incubated in 50 μl of a biotinylated anti-IgM antibody (clone eB121-15F9, eBioscience, 200-fold dilution) on ice for 20 min. The cells were then washed three times and stained by 50 μl of streptavidin-conjugated PE (eBioscience, 600-fold dilution) on ice for 20 min.

### Adoptive transfer of CD4^+^ T cells

Fifteen million spleen cells from DO11.10 mice, containing approximately 3×10^6^ OVA-specific CD4^+^ T cells, were transferred i.v. in 0.2 ml PBS into BALB/c recipient mice one day before immunization with antigen. When CFSE-labeled OVA-specific CD4^+^ T cells were used, CD4^+^ T cells were isolated from DO11.10 splenocytes by magnetic activated cell sorting (MACS) using anti-CD4-conjugated magnetic beads (Miltenyi Biotec, Bergisch Gladbach, Germany) according to manufacturer’s instructions. OVA-specific CD4^+^ cells were then labeled by CFSE (Life technologies) as described previously [28] and 3×10^6^ cells were transferred i.v. in 0.2 ml PBS. Mice were immunized the day after transfer.

### Statistical analysis

Statistical differences between groups were determined by two-tailed Student’s t-test with Prism 5.0d (GraphPad Software, La Jolla, CA USA). Statistical significance levels were set at: ns = p > 0.05; * = p < 0.05; ** = p < 0.01; *** = p < 0.001.

## Results

### IgM from Cμ13 mice cannot lyse SRBC

Previous analyses have shown that IgM from Cμ13 mice cannot form direct hemolytic plaques or cause deposition of C3 on SRBC *in vitro* [15]. Here, SRBC-specific IgM from Cμ13 and BALB/c mice, with the same concentrations (1.74 mg/ml as determined by OD_280 nm_ assuming that an OD of 1.5 corresponds to 1 mg/ml), were tested in hemolysis and hemagglutination assays. IgM preparations from both strains had a hemagglutination titer of 1:128. WT IgM had a hemolytic titer of 1:128 whereas the hemolytic titer of Cμ13 IgM was undetectable (< 1:4), thus confirming the inability of mutant IgM to activate complement.

### IgM from WT but not Cμ13 mice causes rapid deposition of complement on SRBC in the blood

**Figure 1:**
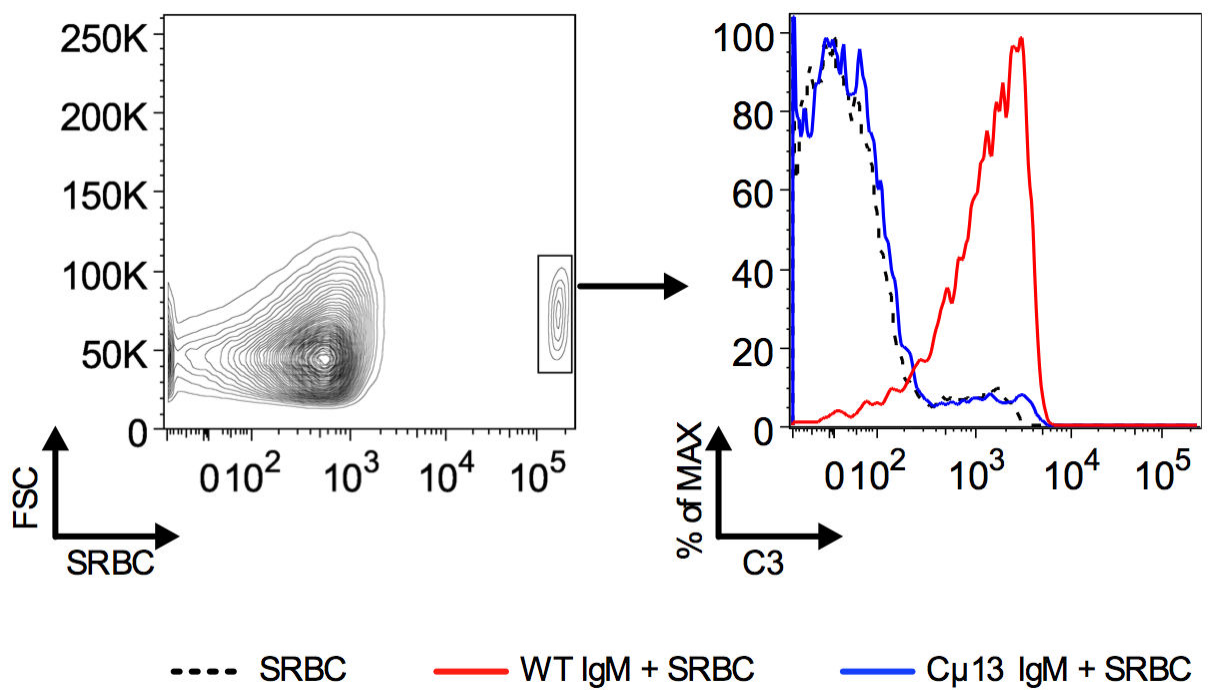
WT IgM induces rapid deposition of C3 on SRBC in the blood. BALB/c mice were immunized i.v. with IgM anti-SRBC (0.2 ml of a solution with HA titer 1:32) from WT BALB/c (WT IgM; n = 2) or Cµ13 mice (Cµ13 IgM; n = 2). Thirty minutes later, 5×10^8^ SRBC were administered. Mice given 5×10^8^ SRBC alone were used as controls (n=2). Peripheral blood samples were taken 1 min after immunization and immediately mixed with 1 µl lepirudin (50 mg/ml). Rabbit IgG anti-SRBC was used to identify SRBC in the blood (left panel). The deposition of C3 on SRBC in the blood from mice immunized with SRBC alone (black) or together with WT IgM (red) or Cµ13 IgM (blue) was analyzed (right panel). Representative of three independent experiments.

WT IgM induces deposition of C3 on SRBC *in vitro* [15]. Here the *in vivo* deposition of C3 on SRBC in the blood was investigated. SRBC-specific IgM from WT or Cμ13 mice was administered to BALB/c mice, followed by SRBC 30 min later. One minute after immunization with SRBC the mice were bled and SRBC were identified in peripheral blood using rabbit anti-SRBC (Figure 1, left panel). WT IgM induced massive deposition of C3 on SRBC after 1 min whereas Cμ13 IgM did not induce any deposition above that seen in mice immunized with SRBC alone (Figure 1, right panel). Interestingly, significant C3 deposition was seen already after 10 s in the WT IgM-group (not shown), but was more pronounced after 1 min.

### IgM from Cμ13 mice cannot enhance antibody responses

**Figure 2:**
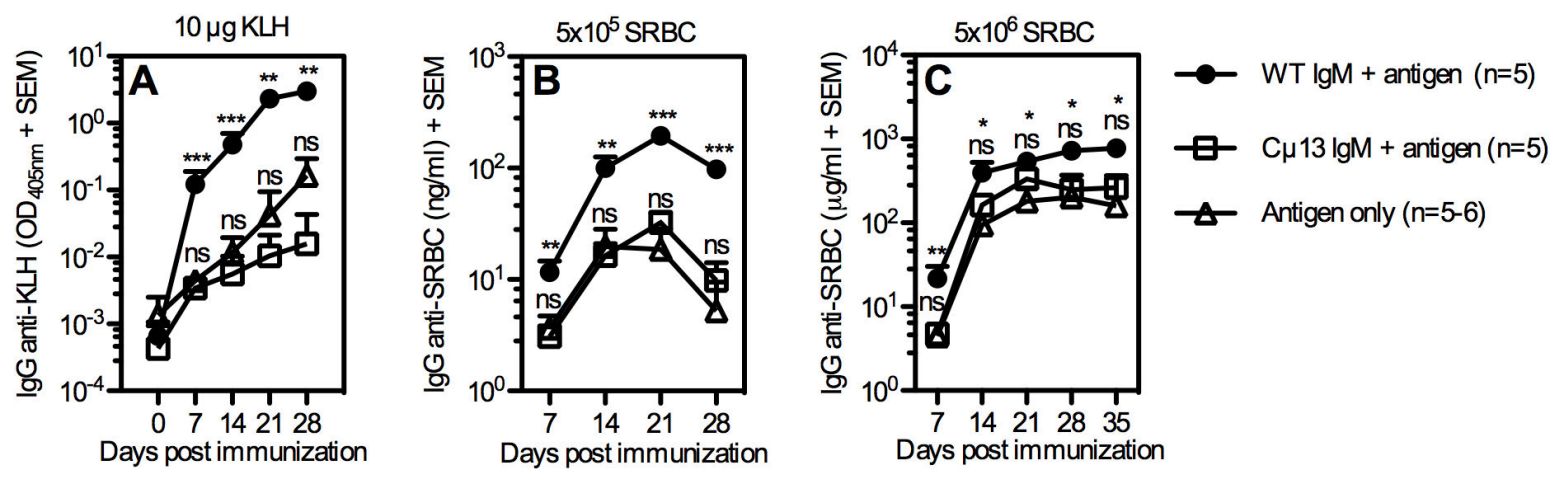
IgM from Cμ13 mice cannot enhance antibody responses. BALB/c mice were immunized i.v. with WT or Cµ13 IgM anti-KLH (50 µg/mouse) or anti-SRBC (0.2 ml of a solution with HA titer 1:8). Thirty minutes later, 10 µg KLH, 5×10^5^ or 5×10^6^ SRBC were administered. Mice immunized with only antigen or IgM were used as controls. All groups were bled at indicated time points and sera were screened for IgG anti-KLH (A) or IgG anti-SRBC (B, C). Antibody responses of mice immunized with specific IgM only were much lower than that of mice immunized with antigens alone (data not shown). Data represent three (A, B) or two (C) experiments. Statistical comparison is shown between mice given antigen alone and mice given antigen together with WT IgM (above) or Cµ13 IgM (below). ns = not significant; * = p < 0.05; ** = p < 0.01; *** = p < 0.001.

To investigate whether Cμ13 IgM can enhance antibody responses, antigen-specific IgM from WT or Cμ13 mice was administered i.v. 30 minutes prior to i.v. immunization with the respective antigens. Control mice were immunized with antigen alone. WT IgM anti-KLH induced significant enhancement of antibody responses whereas the same amount of Cµ13 IgM anti-KLH was a poor enhancer (Figure 2A). Similar results were seen when WT IgM or Cµ13 IgM anti-SRBC were given together with two different doses of SRBC (Figure 2B,C). Thus, IgM obtained from Cµ13 mice was unable to enhance specific antibody responses while the same doses of IgM from BALB/c mice enhanced efficiently.

### IgM from WT mice enhances the germinal center response

**Figure 3:**
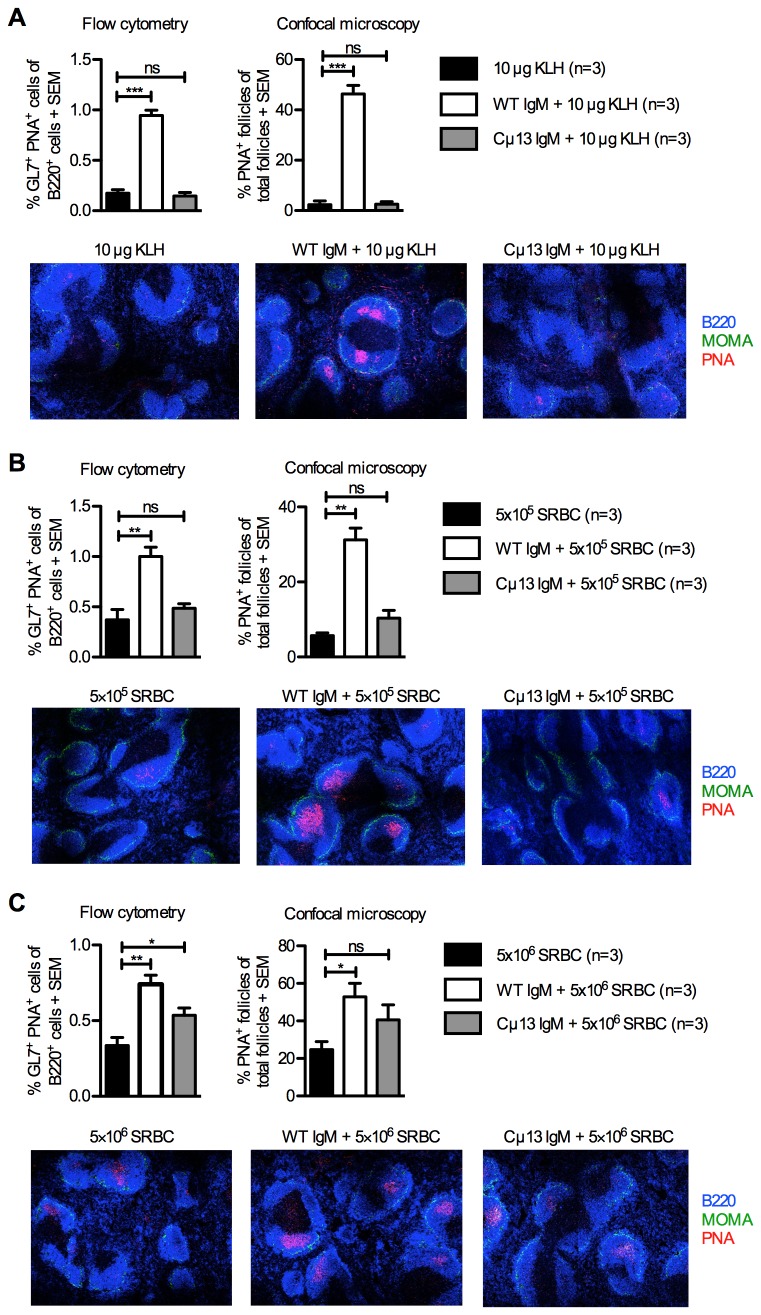
IgM from BALB/c mice enhances germinal center responses. On day 0, BALB/c mice were immunized i.v. with WT or Cµ13 IgM specific for KLH (50 µg/mouse) or SRBC (0.2 ml of a solution with HA titer 1:32) 30 min before 10 µg KLH (A), 5×10^5^ (B) or 5×10^6^ SRBC (C) were administered via the same route; controls received antigens or specific IgM alone. Spleens were harvested on day 10. Splenocytes from half of each spleen were analyzed by flow cytometry; germinal center B cells were gated as GL7^+^PNA^+^ amongst B220^+^ cells (Figure S1) and the percentages of germinal center B cells were quantified (A-C, upper left panels). The other halves of the spleens were sectioned, stained with anti-B220 (blue), anti-MOMA (green) and PNA (red), and analyzed for number of PNA^+^ germinal centers in B cell follicles by confocal microscopy (A-C, upper right panels); each image is a representative area (1725 µm × 1295 µm) for 2-3 whole sections with original magnification ×10 (A-C, lower panels). Germinal center responses of mice immunized with specific IgM alone were always lower than the responses of mice immunized with antigens alone (not shown). Data are representative of two experiments with each antigen dose. ns = not significant; * = p < 0.05; ** = p < 0.01.

Next, we compared germinal center responses in mice immunized with antigen alone or with WT or Cµ13 IgM 30 min prior to antigen. Specific WT IgM but not Cµ13 IgM administered together with KLH induced an increased percentage of GL7^+^PNA^+^ germinal center B cells in the animals as compared to KLH alone (Figure 3A, upper left panel, Figure S1). This result was paralleled by an increased percentage of PNA^+^ follicles containing germinal centers in mice given WT IgM and antigen (Figure 3A, upper right panel and representative confocal tile-scan images). Similar results were obtained when SRBC was used as antigen (Figure 3B,C). Mice given specific Cµ13 IgM together with the lower dose of SRBC did not have an enhanced germinal center reaction as compared to mice given SRBC alone (Figure 3B). Mice given Cµ13 IgM together with a ten-fold higher dose of SRBC had a slightly enhanced germinal center reaction although the increase was less pronounced than with WT IgM (Figure 3C). In summary, WT IgM always enhanced formation of germinal centers. Cµ13 IgM usually had no significant effect but a slight enhancement could be seen against the highest dose of SRBC.

### IgM from WT and Cμ13 bind equally well to FcμR

**Figure 4:**
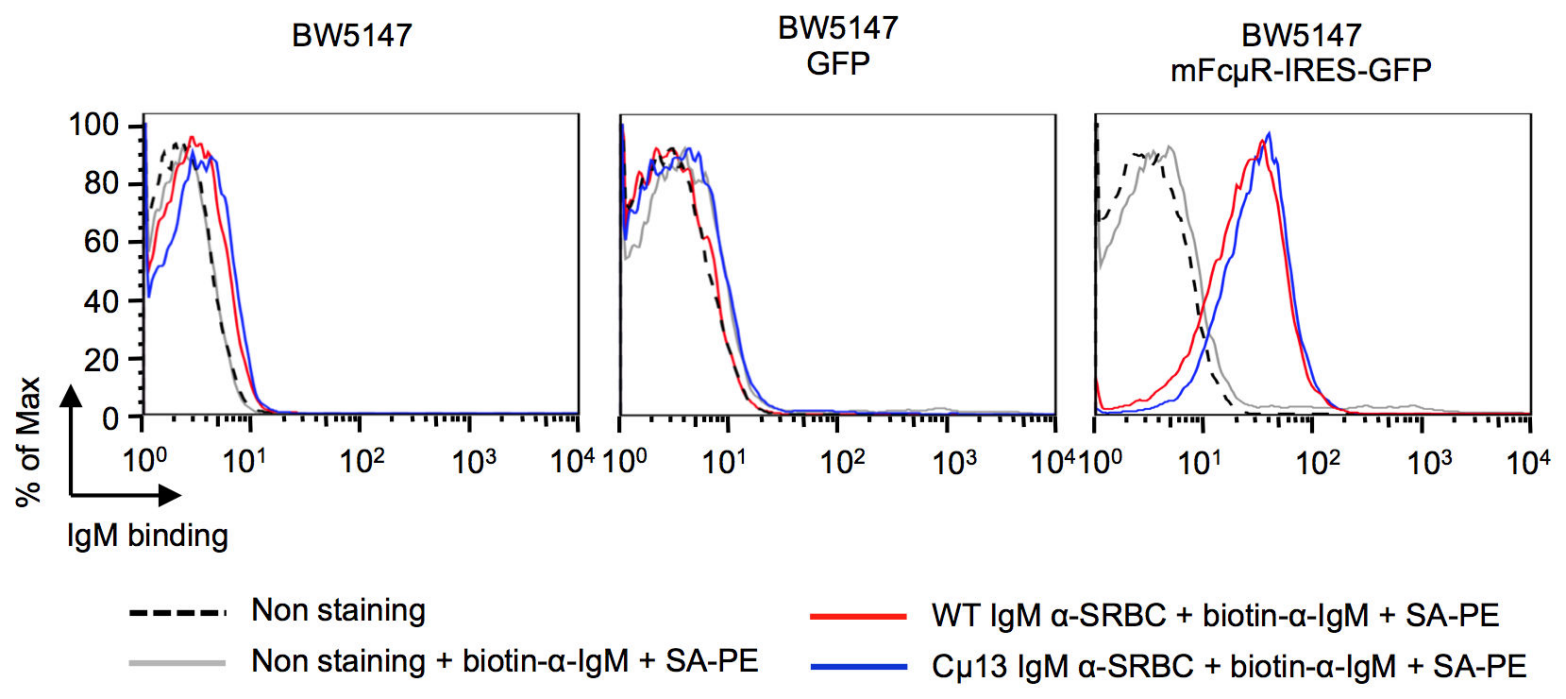
IgM from BALB/c and Cμ13 bind equally well to FcμR. BW5147 cells expressing mFcμR-IRES-GFP (right panel) or GFP alone (middle panel), as well as virus-nontransduced BW5147 (left panel) cells were analyzed for binding to SRBC-specific WT IgM (red) or Cμ13 IgM (blue) antibodies. The unstained BW5147 (black) and BW5147 stained with biotinylated anti-IgM and SA-PE (grey) are shown as controls.

To investigate whether the point mutation of the Cμ13 IgM resulted in impaired ability to bind to the murine FcμR, BW5147 cells stably expressing mFcμR-IRES-GFP or GFP alone, were established (Figure S2). Subsequently, the binding of WT and Cμ13 IgM to these cells was analyzed in flow cytometry showing that both types of IgM antibodies bound equally well to cells expressing mFcμR-IRES-GFP (Figure 4, right panel, red and blue lines). Neither WT nor Cμ13 IgM bound to cells expressing GFP alone (middle panel) or to virus-nontransduced cells (left panel). These results clearly demonstrate that the Cμ13 IgM antibodies are able to bind to mouse FcμR as efficiently as are WT IgM antibodies. 

### Proliferation of antigen-specific CD4^+^ T cells is not enhanced by specific IgM

**Figure 5:**
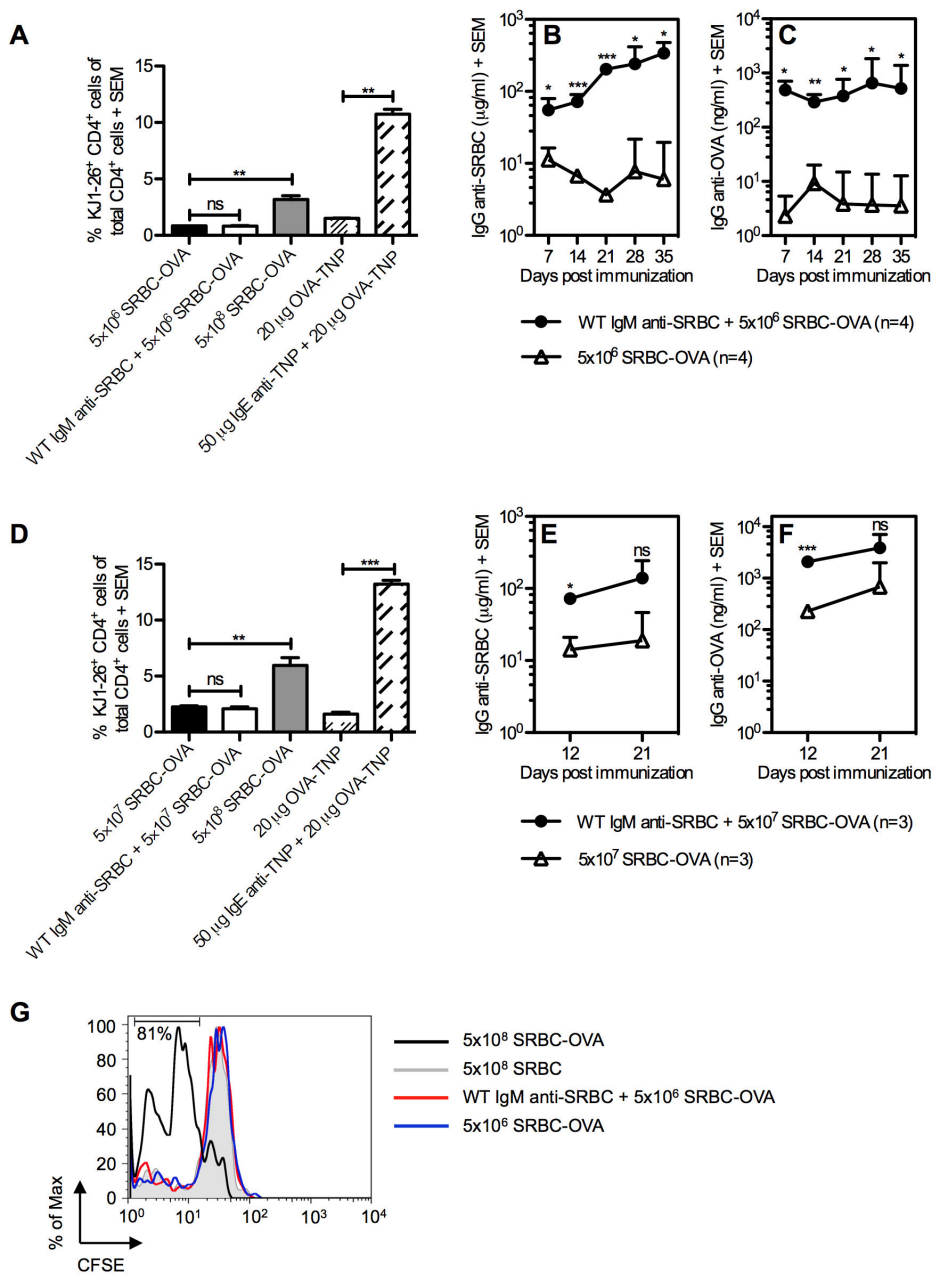
Proliferation of antigen-specific CD4^+^ T cells is not enhanced by specific IgM. On day 0, 15×10^6^ splenocytes from DO11.10 mice (A-F) or 3×10^6^ CFSE-labeled CD4^+^ DO11.10 cells (G) were adoptively transferred to BALB/c mice. On day 1, recipients were immunized with 5×10^6^ (A-C, G) or 5×10^7^ SRBC-OVA (D-F) alone or with WT IgM anti-SRBC (0.2 ml of a solution with a HA titer 1:8) 30 min before administration of SRBC-OVA. Mice immunized with 5×10^8^ SRBC-OVA or 20 μg OVA-TNP with or without 50 μg IgE anti-TNP were used as positive controls. Mice immunized with 5×10^7^ or 5×10^8^ unconjugated SRBC were used as negative controls. On day 4, i e three days after administration of antigen, spleens from 2-3 mice in each group were harvested and analyzed by flow cytometry (A,D,G). OVA-specific T cells were identified as KJ1-26^+^CD4^+^ cells (A, D) or KJ1-26^+^ cells (G). Percentages of KJ1-26^+^CD4^+^ cells in negative control mice were always lower than in mice immunized with OVA-TNP alone (not shown). CFSE intensity of KJ1-26^+^ cells was measured three days after immunization by flow cytometry (G). Representative histograms of 2-3 mice in each group are shown. Three to four mice per group were left for weekly blood sampling and sera were screened for IgG anti-SRBC (B,E) and IgG anti-OVA (C,F) in ELISA. Data represent one (A-C,G) or three (D-F) independent experiments. ns = not significant; * = p < 0.05; ** = p < 0.01; *** = p < 0.001.

Both SRBC and KLH are T-dependent antigens and it was of interest to find out whether IgM in parallel with upregulating humoral responses also affected T helper cell responses. The role of IgM in CD4^+^ T cell proliferation was investigated *in vivo*, using a system where BALB/c mice were adoptively transferred with splenocytes containing transgenic OVA-specific CD4^+^ T cells. One day after transfer, mice were given WT IgM anti-SRBC and SRBC-OVA or SRBC-OVA alone. IgM-mediated enhancement is non-epitope specific [6,9,11], and as expected the serum levels of both IgG anti-SRBC and IgG anti-OVA were enhanced (Figure 5B,C,E,F). IgM-mediated enhancement is less efficient when higher antigen doses are used [4], probably explaining why the enhancement was less significant using 5×10^7^ (Figure 5E,F) than 5×10^6^ SRBC-OVA (Figure 5B,C). Nevertheless, the higher dose was included since T cell responses to 5×10^6^ SRBC-OVA were very low.

**Figure 6:**
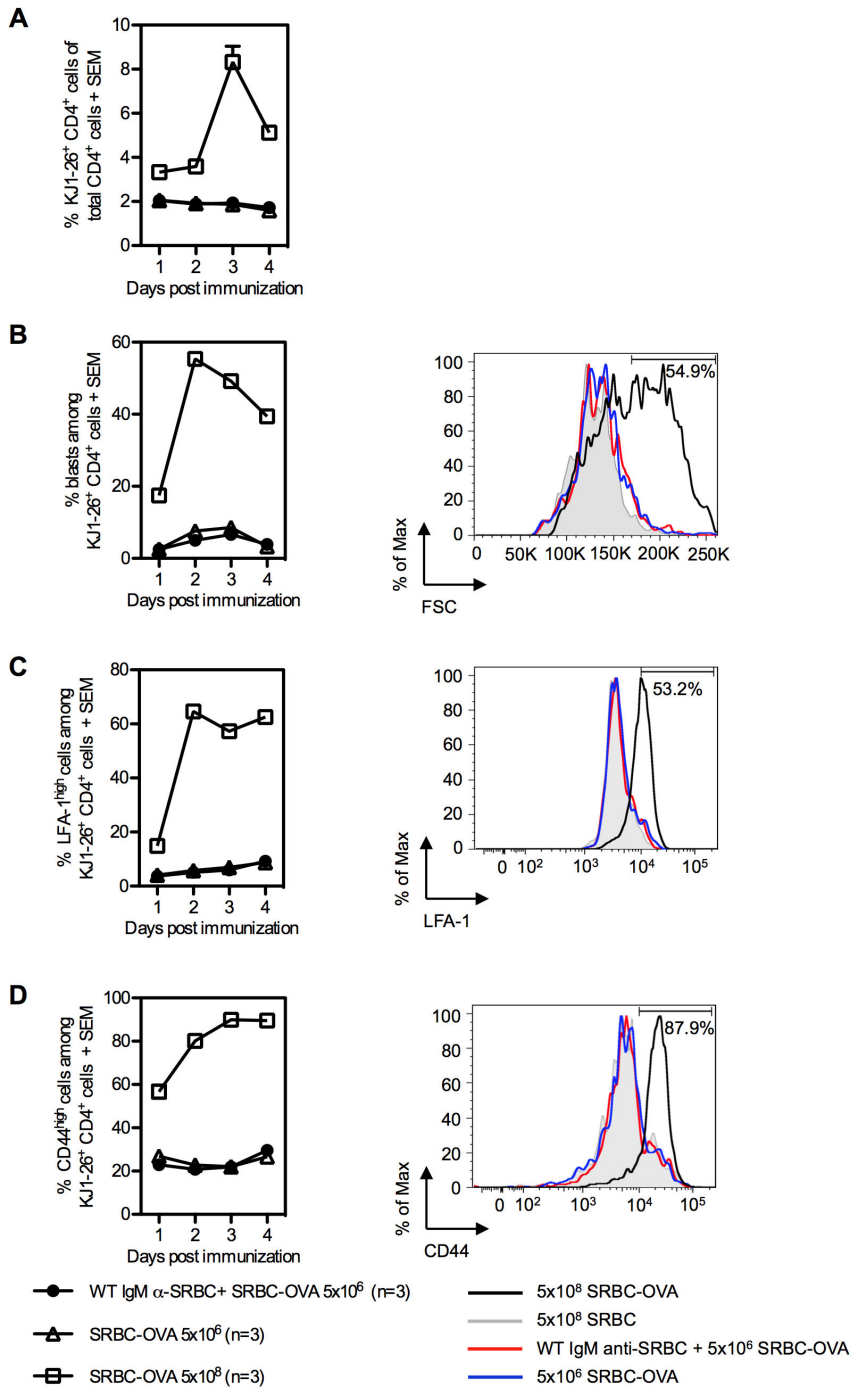
Activation of antigen-specific CD4^+^ T cells is not enhanced by specific IgM. BALB/c mice were adoptively transferred with 15×10^6^ splenocytes from DO11.10 mice. The next day, mice were immunized with 5×10^6^ SRBC-OVA alone or together with WT IgM anti-SRBC (0.2 ml of a solution with HA titer 1:8), 5×10^8^ SRBC-OVA (positive controls) or 5×10^8^ unconjugated SRBC (negative controls). One, 2, 3, and 4 days after immunization, spleens from 2-3 mice per group were harvested and analyzed by flow cytometry. Kinetics of KJ1-26^+^CD4^+^ cell expansion (A), percentages of blasts among KJ1-26^+^CD4^+^ cells (B, left panel) and expression of surface markers LFA-1 (C, left panel) and CD44 (D, left panel) on KJ1-26^+^CD4^+^ cells were quantified. Numbers in histograms indicate the percentages of blasts, LFA-1^high^ and CD44^high^ cells among KJ1-26^+^CD4^+^ cells in the positive controls. Representative histograms (B-D, right panels) of each group immunized with 5×10^8^ SRBC-OVA (black), 5×10^8^ unconjugated-SRBC (grey), 5×10^6^ SRBC-OVA alone (blue) or together with WT IgM anti-SRBC (red) are shown for day 3. SEM were frequently too small to be seen in the figures.

In spite of the effect on antibody responses, no enhancement of the proliferation of OVA-specific CD4^+^ cells was induced by IgM, neither at the peak of the response three days after immunization (Figure 5A,D) nor at any other time points (Figure 6A). Positive controls, consisting of an optimal dose of SRBC-OVA (5×10^8^) or IgE-anti-TNP/OVA-TNP complexes (known to induce marked CD4^+^ T cell proliferation in this system [28]), both induced proliferation of OVA-specific CD4^+^ T cells demonstrating that CD4^+^ T cells were indeed able to proliferate in this system (Figure 5A,D, Figure 6A). 

In the experiments described above, T cell proliferation was assumed to explain the increased numbers of OVA-specific CD4^+^ T cells. To exclude that this increase was merely an effect of enhanced migration of T cells to the spleen, proliferation was tested in an alternative approach. CD4^+^ T cells from DO11.10 mice were isolated, CFSE-labeled and adoptively transferred to BALB/c mice one day prior to immunization with WT IgM anti-SRBC and 5×10^6^ SRBC-OVA or 5×10^6^ SRBC-OVA alone. Positive controls were immunized with 5×10^8^ SRBC-OVA and negative controls with 5×10^8^ unconjugated SRBC. Three days after immunization, 81% of the CFSE-labeled cells in the positive controls had divided. No difference in cell division between mice immunized with IgM and SRBC-OVA and mice immunized with either SRBC-OVA alone or unconjugated SRBC could be seen (Figure 5G). Thus, we could find no evidence of IgM-mediated enhancement of T-cell proliferation using antigen doses which led to significant enhancement of antibody responses (Figure 5B,C).

To follow the kinetics and activation profile of OVA-specific CD4^+^ cells, BALB/c mice were adoptively transferred with splenocytes from DO11.10 mice and immunized one day later. Spleens were harvested after 1, 2, 3, or 4 days and tested for number of OVA-specific T cells, blast transformation and expression of markers for T cell activation (LFA-1 and CD44) (Figure 6). KJ1-26^+^CD4^+^ (OVA-specific) T cells from mice immunized with 5×10^8^ unconjugated SRBC had low levels of blast transformation, LFA-1 and CD44 expression. (Figure 6, grey areas in the histograms). KJ1-26^-^CD4^+^ (unspecific) T cells from mice immunized with 5×10^8^ SRBC-OVA had equally low levels of these parameters (not shown). OVA-specific T cells in mice immunized with 5×10^8^ SRBC-OVA responded with increased numbers in the spleen, blast transformation, LFA-1, and CD44 expression (Figure 6A-D). Interestingly, groups immunized with 5×10^6^ SRBC-OVA, with or without WT IgM anti-SRBC, both had low responses and there was no evidence of enhancement in the IgM-groups (Figure 6A-D). In summary, SRBC-specific IgM administered with SRBC-OVA is capable of enhancing the antibody-responses to SRBC and OVA (Figure 5B,C,E,F) but there is no evidence of an enhancing effect by IgM on OVA-specific CD4^+^ T cells as measured by T cell numbers (Figure 5A,D, Figure 6A), cell divisions (Figure 5G), blast cell transformation (Figure 6B) or upregulation of the activation markers LFA-1 and CD44 (Figure 6C,D).

## Discussion

We have addressed the question of whether the immunoenhancing effect of antigen-specific IgM relates to its ability to activate complement or to its ability to bind to FcμR. IgM with a point mutation in the Cμ chain, shown to abrogate the ability to activate complement, retained the ability to bind to the murine FcμR (Figure 4) but lost its ability to enhance antibody and germinal center responses and to induce *in vivo* deposition of C3 on SRBC (Figures 1-3). These observations show that complement-activation, and not FcμR binding, is required for enhancement of antibody responses by passively administered specific IgM. This confirms previous work [10,11] and emphasizes the importance of classical pathway complement activation for antibody responses [18]. 

An interesting question is how this can be reconciled with the ability of natural IgM as well as FcμR to potentiate antibody responses [19-22], especially since the enhancement by natural IgM does not seem to require complement activation [15]. The antigens studied herein are all administered i.v. and are therefore transported to the spleen, where the initial antibody responses take place. MZ B cells reside in spleen, but not in lymph nodes, and express high levels of FcμR [21,22] and complement receptors 1 and 2 (CR_1/2_). They continuously shuttle between the MZ and the follicles and can deposit antigen onto FDC, a process often requiring binding of antigen to CR_1/2_ on the MZ B cells [33-36]. However, it seems plausible that MZ B cells can also capture antigen via FcμR, which in naive mice are most likely saturated with natural IgM. This would enable them to deliver non-complement-opsonized antigen to FDC and perhaps explain the complement-independent immunoenhancing effects of natural IgM and FcμR. 

Another interesting question is how specific IgM, in what clearly seems to be a complement dependent procedure, operates to enhance antibody responses. Lack of complement factors C1q, C2, C3, and C4, as well as lack of CR_1/2_, results in impaired antibody responses (reviewed in 18). Since the ligands for CR_1/2_ are split products of complement factor C3, it is likely that the effects of complement in facilitating antibody responses is largely dependent on CR_1/2_. In mice, these receptors are derived from the same gene, Cr2, by alternative splicing [37]. They are expressed on B cells and FDC [38,39], which gives them ample opportunity to influence immune responses. Studies of IgM-mediated enhancement in bone marrow chimeric mice (expressing CR_1/2_ on FDC, on B cells, on both cell types or on none of the cell types) showed that an optimal antibody response to IgM-SRBC complexes required that both FDC and B cells express the receptors, but that expression on FDC was of major importance [8]. 

Presence of antigen on FDC is thought to be an important step in the development of germinal centers, class switch recombination and somatic hypermutation [40]. Therefore, the observation that IgM increases the size and number of germinal centers (Figure 3) as well as the kinetics of their induction [35], suggests that IgM increases the capture of antigen on CR_1/2_^+^ FDC in B cell follicles. Cμ13 IgM has a markedly reduced capacity to enhance the germinal center reaction, illustrating the complement dependence of the process. This is in line with other studies showing that deficiency of C3, C4, or CR_1/2_ leads to impaired development of germinal centers [41-43]. Whereas germinal center development is an indirect measure of follicular antigen deposition, direct detection of antigen, administered with specific IgM, in splenic follicles has also been reported. Bovine serum albumin (BSA) and virus-like particles, administered with IgM, have been detected directly in follicles, although it was not determined whether IgM enhanced the antibody responses in parallel with enhancing the deposition of antigen [35,36]. In one study, IgM-mediated enhancement of antibody responses and localization of radioactively labeled SRBC to the spleen were shown to correlate, but whether the antigen reached follicles could not be determined [5]. SRBC and KLH are interesting antigens in this context since they represent large structures against which IgM has been shown to enhance antibody responses. To our knowledge, these antigens have never been detected in splenic follicles and we tried to visualize them after labeling with biotin, PKH26, Alexa Fluor 647, or CFSE, or after injection in native form followed by staining of the sections with hyperimmune rabbit anti-SRBC. However, antigen could not be detected in follicles at any of the time points between 10 min and 24 h that were tested. Both SRBC and KLH were seen in the MZ during the first hour after injection, but the antigen amount decreased within the next 4 h. The reason for the lack of detectable antigen in follicles is not understood. One possibility is that these antigens are rapidly internalized by FDC [44] but they may also be rapidly degraded by other cells and therefore impossible to visualize.

B cells can hypothetically use CR_1/2_ in several ways to facilitate antibody responses. 

*In vitro*, co-crosslinking of CR_1/2_ and the B cell receptor lowers the threshold for B cell activation [45]. We cannot exclude that this mechanism contributes to the effects of IgM, but since only occasional and low responses were seen when B cells alone expressed CR_1/2_, it is probably of minor importance [8]. Another mechanism shown to operate *in vitro*, is that B cells take up opsonized antigen via CR_1/2_ and present it efficiently to T helper cells [46]. The observation that IgM anti-SRBC administered with SRBC-OVA did not increase the activation of OVA-specific T cells, at least not when assayed by any of the parameters used herein, although it efficiently enhanced antibody responses (Figures 5 and 6), argues against this idea. A divergence between the regulation of T- and B-cell responses has been seen in other situations involving complement. IgG3 enhances antibody responses against protein antigens in a complement dependent manner [26] but does not enhance CD4^+^ T cell proliferation [47] and mice lacking CR_1/2_, C4, or C3 have impaired antibody responses but normal T helper cell responses [24,43,48,49]. FDC present non-processed antigen to B cells, but do not express MHC molecules and therefore cannot present antigen to T cells. Thus, should IgM enhance antibody responses by increasing the deposition of antigen on FDC, no effects on T cell proliferation would be expected. A third possibility for B cells to affect antibody responses is by transporting IgM-antigen-complement complexes from the MZ to the follicles as discussed above [33-36]. MZ B cells express high levels of CR_1/2_ and deliver complexes of specific IgM and antigen to FDC [35]. We find it likely that enhancement of antibody responses by specific IgM starts with a rapid deposition of C3 on the antigen in the blood. When these immune complexes reach the marginal zone, they are captured by CR_1/2_^+^ MZ B cells and transported into follicles where they are efficiently deposited on CR_1/2_^+^ FDC. This scenario would explain the requirement for CR_1/2_ both on B cells and FDC for optimal IgM-mediated enhancement [8]. In situations where no specific IgM is available, as in a primary antibody response, MZ B cells can capture antigen via natural IgM bound to FcμR, and transport it into follicles through the shuttling mechanism. Importantly, antigen will not be captured, endocytosed and presented to B cells by FDC unless it is opsonized with complement since FDC-mediated capture takes place via CR_1/2_. How the classical pathway-dependent complement activation takes place in situations where neither specific IgM nor complement-activating natural IgM is available, is an enigma [15].

## Supporting Information

Figure S1 **Gating strategy for germinal center B cells in flow cytometry.** Lymphocytes were first gated according to forward- and side-scatter (left panel). B cells were then gated as B220^+^ cells (middle panel). Germinal center B cells were gated as GL7^+^ PNA^+^ cells among all B220^+^ cells (right panel).
(TIF)

Figure S2 **Preparation of mouse FcμR (mFcμR) stable transductants.** The BW5147 mouse T cells were transduced with retrovirus expressing mFcμR-IRES-GFP or GFP alone and the GFP^+^ cells were sorted. Virus-nontransduced BW5147 cells were included as a control. These cells were either left unstained (Non staining), or were stained with an isotype control or the 4B5 anti-mFcμR monoclonal antibody. (A) FACS profiles of GFP vs. FcμR expression. FcμR was only detected on BW5147 cells transduced with mFcμR-IRES-GFP (lower right panel), but not on virus nontransduced BW5147 (upper right panel) or BW5147 expressing GFP alone (middle right panel). (B) Histograms of FcμR expression in nontransduced BW5147 (left panel), and in BW5147 cells expressing GFP alone (middle panel) or mFcμR-IRES-GFP (right panel).
(TIF)
