# Supplementary figures and images for: Complement-Activating IgM Enhances the Humoral but Not the T Cell Immune Response in Mice
Source: PLoS One. 2013 Nov 8;8(11):e81299. doi: 10.1371/journal.pone.0081299 (PMC3826713; doi:10.1371/journal.pone.0081299)

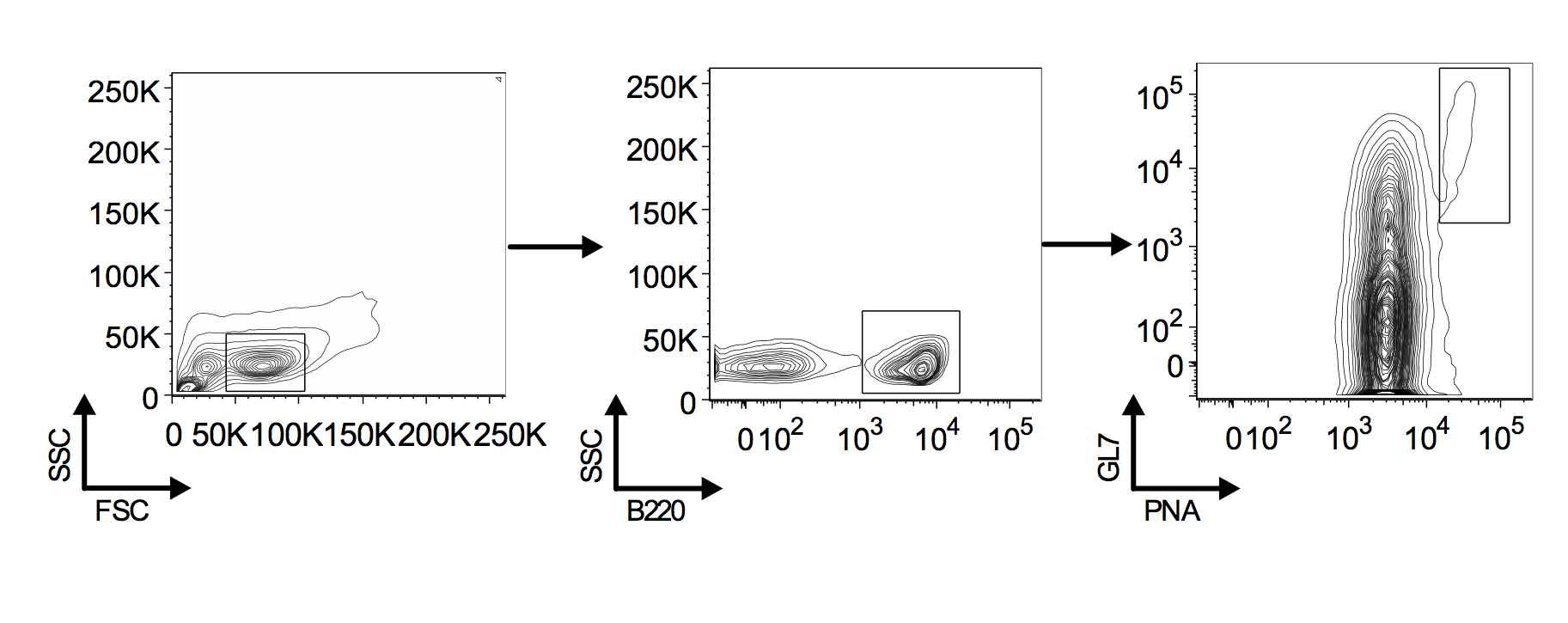

Supplement: Figure S1 — Gating strategy for germinal center B cells in flow cytometry. Lymphocytes were first gated according to forward- and side-scatter (left panel). B cells were then gated as B220+ cells (middle panel). Germinal center B cells were gated as GL7+ PNA+ cells among all B220+ cells (right panel). (TIF) [file pone.0081299.s001.tif]

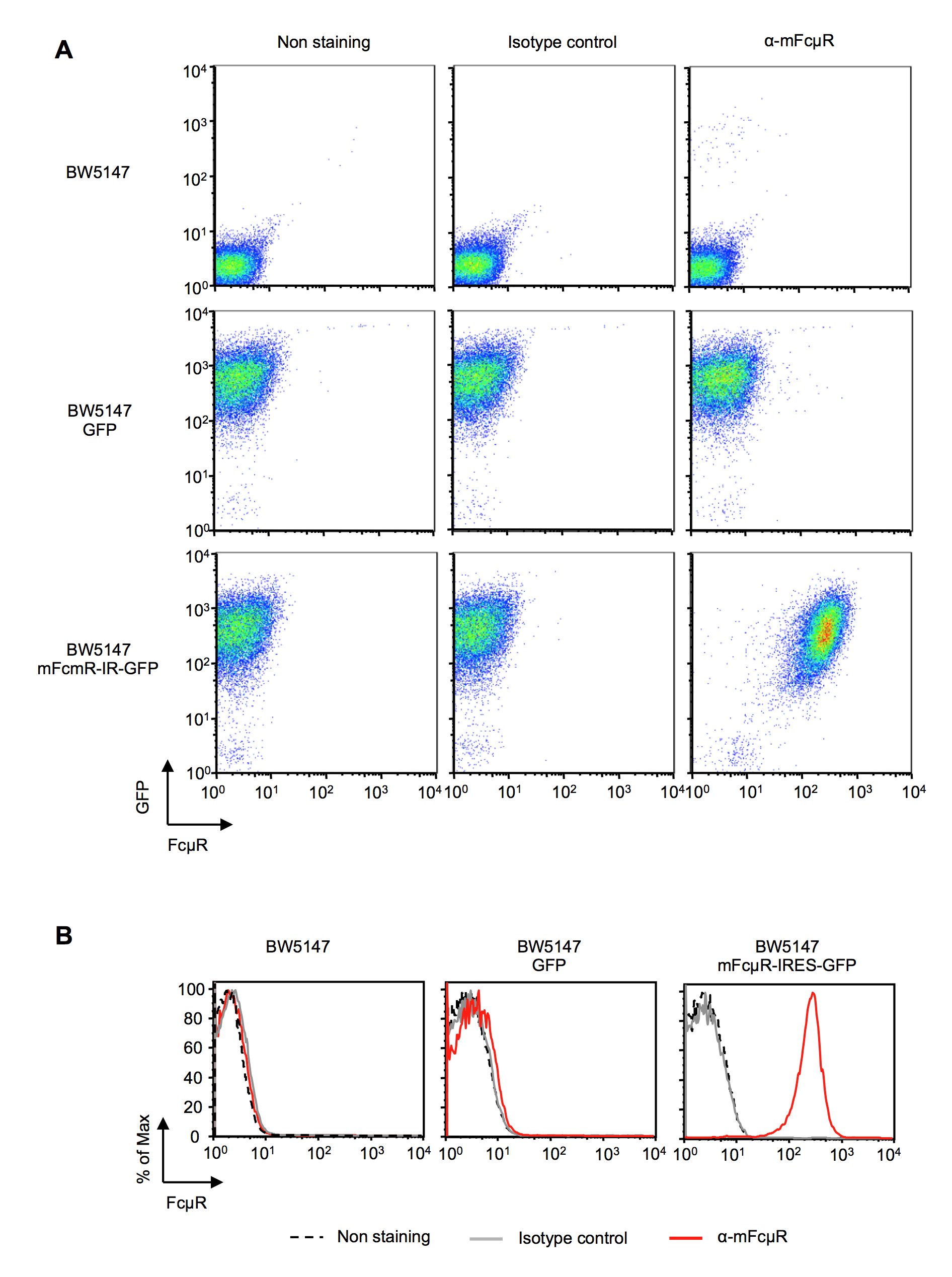

Supplement: Figure S2 — Preparation of mouse FcμR (mFcμR) stable transductants. The BW5147 mouse T cells were transduced with retrovirus expressing mFcμR-IRES-GFP or GFP alone and the GFP+ cells were sorted. Virus-nontransduced BW5147 cells were included as a control. These cells were either left unstained (Non staining), or were stained with an isotype control or the 4B5 anti-mFcμR monoclonal antibody. (A) FACS profiles of GFP vs. FcμR expression. FcμR was only detected on BW5147 cells transduced with mFcμR-IRES-GFP (lower right panel), but not on virus nontransduced BW5147 (upper right panel) or BW5147 expressing GFP alone (middle right panel). (B) Histograms of FcμR expression in nontransduced BW5147 (left panel), and in BW5147 cells expressing GFP alone (middle panel) or mFcμR-IRES-GFP (right panel). (TIF) [file pone.0081299.s002.tif]
